# Supplementary material for: Characterization among and within Sicilian Tetraploid Wheat Landraces by Gluten Protein Analysis for Traceability Purposes
Source: Plants (Basel). 2024 Mar 6;13(5):741. doi: 10.3390/plants13050741 (PMC10935316; doi:10.3390/plants13050741)
Supplement: Supplementary file 1 [file plants-13-00741-s001.zip › plants-2890901-supplementary/Supplementary figures.pdf]

Characterization among and within Sicilian durum wheat landraces by grain storage protein analysis

Samuela Palombieri, Marco Bonarrigo, Silvia Potestio, Francesco Sestili, Bernardo Messina, Giuseppe Russo, Claudia Miceli, Benedetto Frangipane, Marco Genduso, Chiara Delogu, Andreani Lorella and Stefania Masci

Supplementary figures

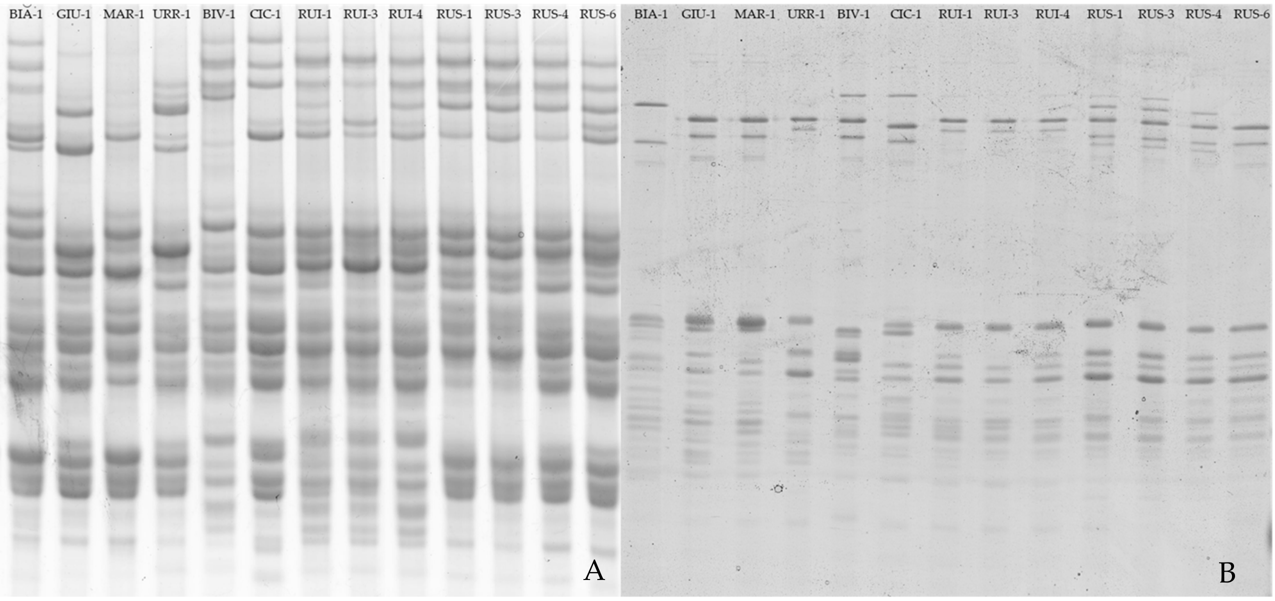

Figure S1. Electrophoretic separation of glutens proteins of the accession bulks. A-PAGE analysis of gliadins fractions (**A**) and on the right SDS-PAGE analysis of glutenins fraction (**B**). Accessions of the varieties analysed: Biancuccia (BIA), Giustalisa (GIU), Martinella (MAR), Urria (URR), Bivona (BIV), Ciciredda (CIC), Russello Ibleo (RUI), Russello (RUS). Each lane corresponds to an accession (Table 3).

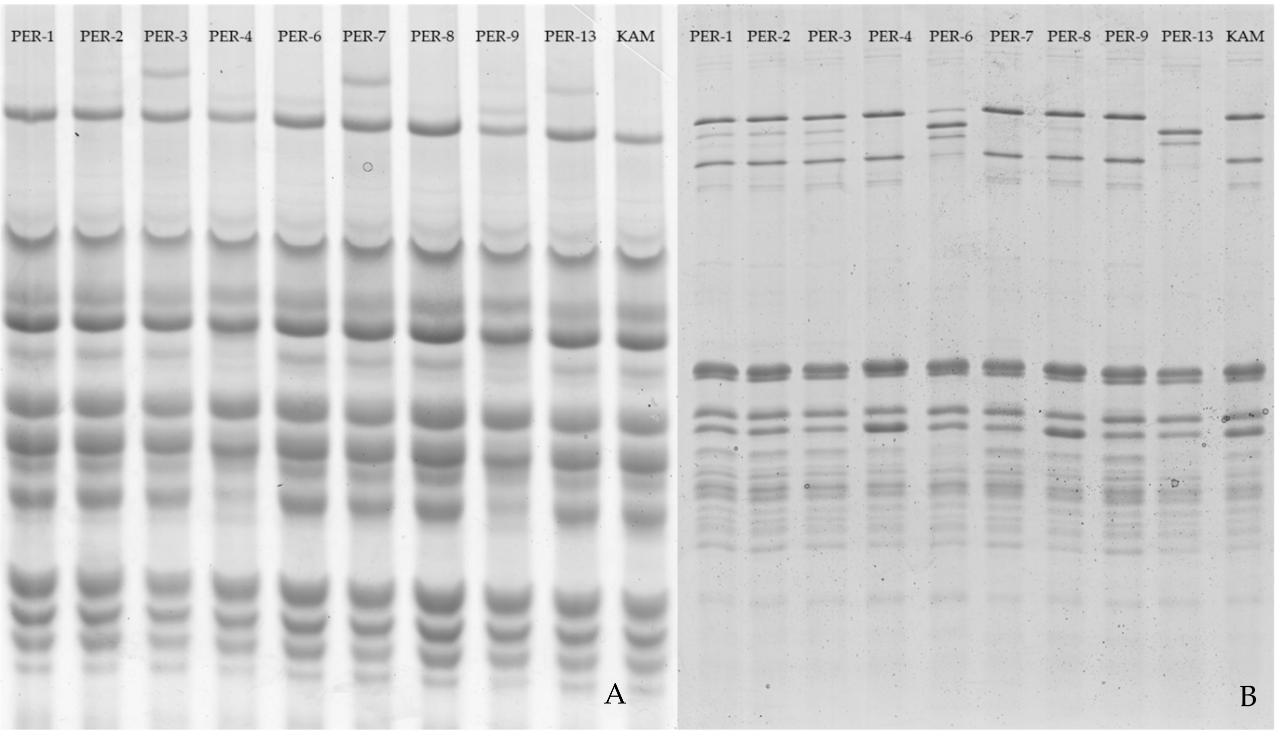

Figure S2. Electrophoretic separation of glutens proteins of the accession bulks. A-PAGE analysis of gliadins fractions (A) and on the right SDS-PAGE analysis of glutenin fraction (B). Accessions of the varieties analysed: Perciasacchi (PER) and Kamut (KAM). Each lane corresponds to an accession (Table 3)..

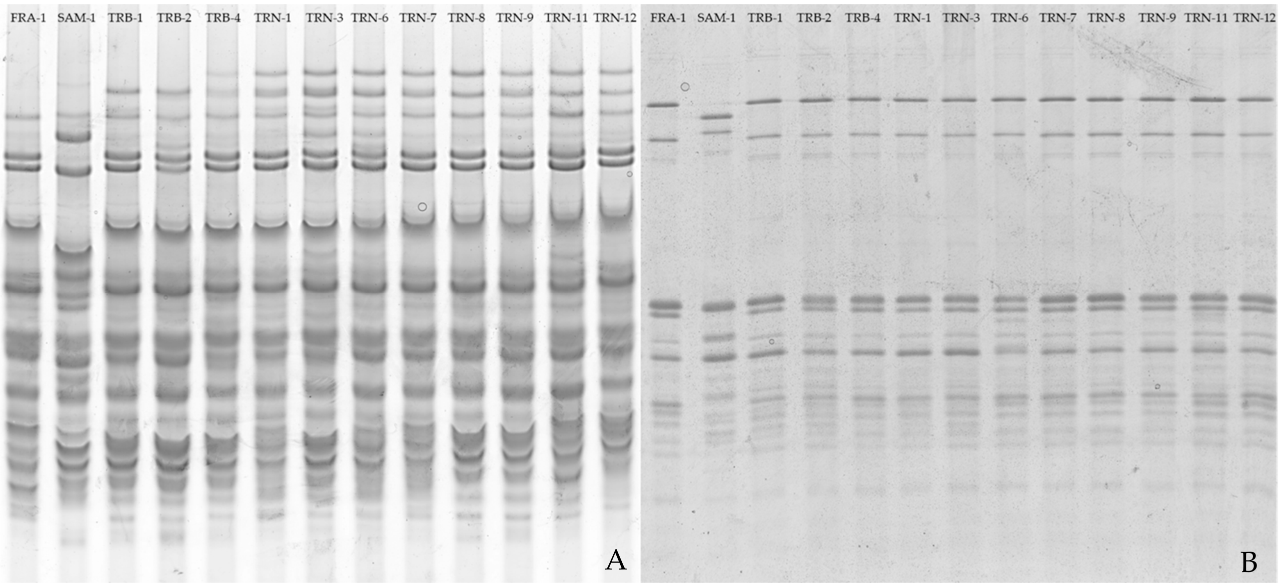

Figure S3. Electrophoretic separation of glutens proteins of the accession bulks. A-PAGE analysis of gliadins fractions (**A**) and on the right SDS-PAGE analysis of glutenin fraction (**B**) of the accessions of the varieties: Francesa (FRA), Sammartinara (SAM), Timilia Reste Bianche (TRB), Timilia Reste Nere (TRN). Each lane corresponds to an accession (Table 3).

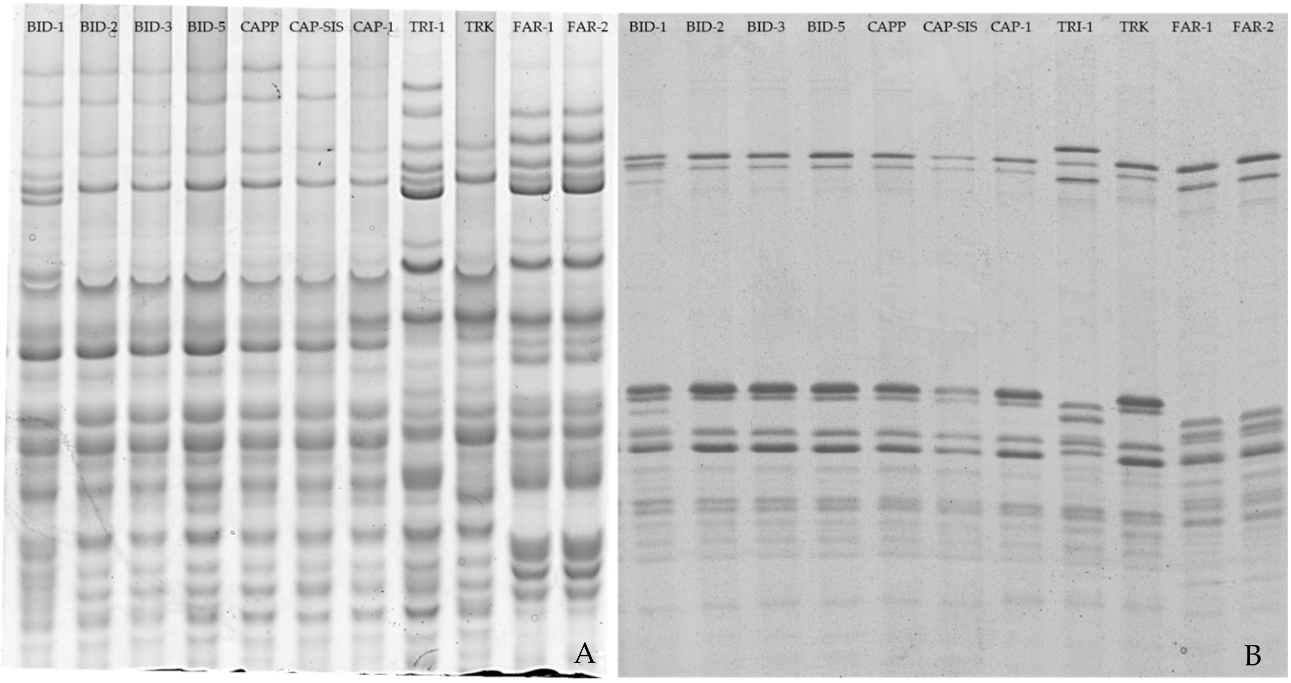

Figure S4. Electrophoretic separation of glutens proteins of the accession bulks. A-PAGE analysis of gliadins fractions (A) and on the right SDS-PAGE analysis of glutenin fraction (B). Accessions of the varieties analysed: Bidì (BID), Cappelli (CAPP), Cappelli – SIS (CAP-SIS), Capeiti 8 (CAP), Tripolino (TRI), Trinakria (TRK), Faricello (FAR). Each lane corresponds to an accession (Table 3).

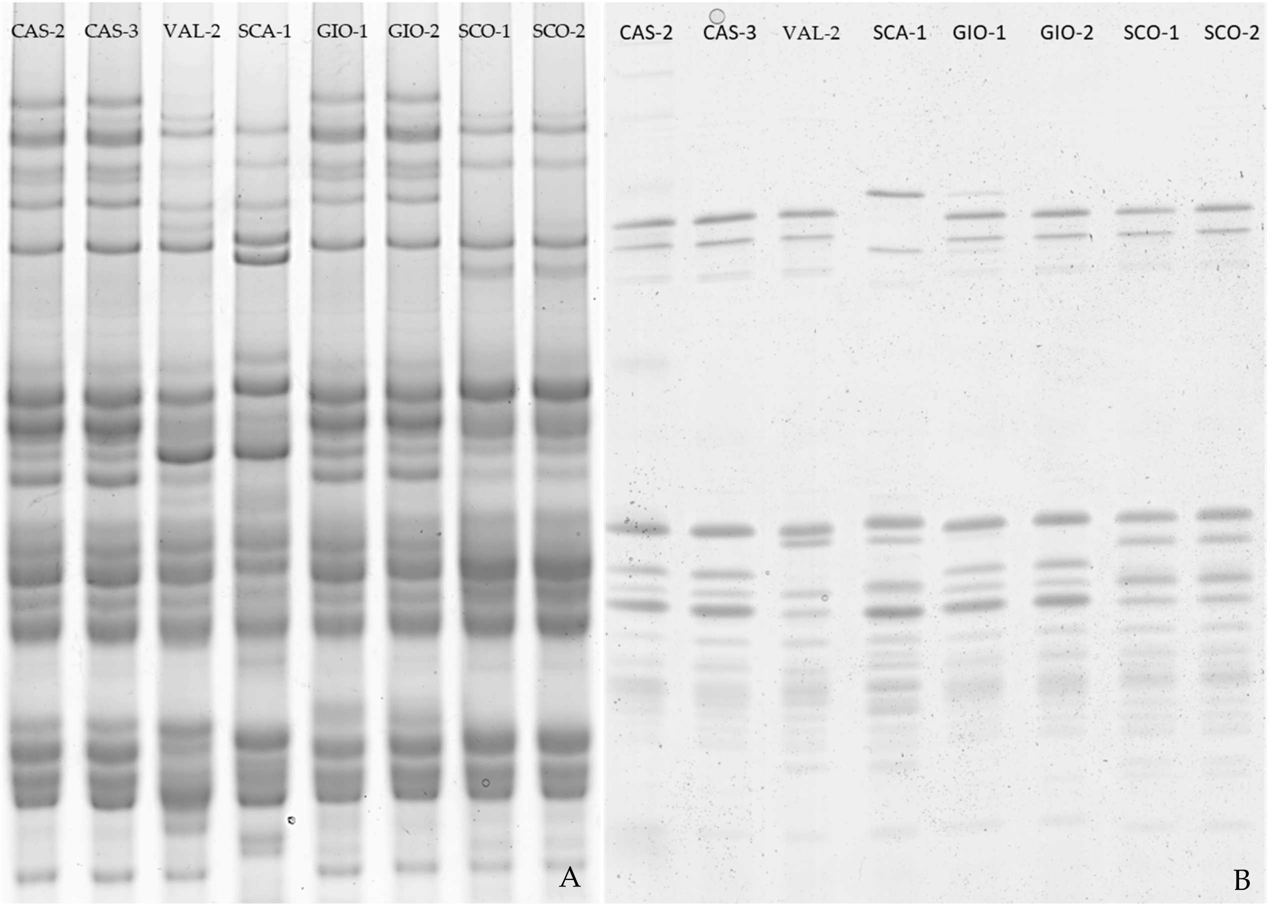

Figure S5. Electrophoretic separation of glutens proteins of the accession bulks. A-PAGE analysis of gliadins fractions (A) and on the right SDS-PAGE analysis of glutenins fraction (B). Accessions of the varieties analysed: Castiglione Glabro (CAS), Vallelunga pubescente (VAL), Scavuzza (SCA), Gioia (GIO), Scorsonera (SCO). Every lane corresponds to an accession (Table 3). On the left A-PAGE analysis of gliadins fractions and on the right SDS-PAGE analysis of glutenin fraction.

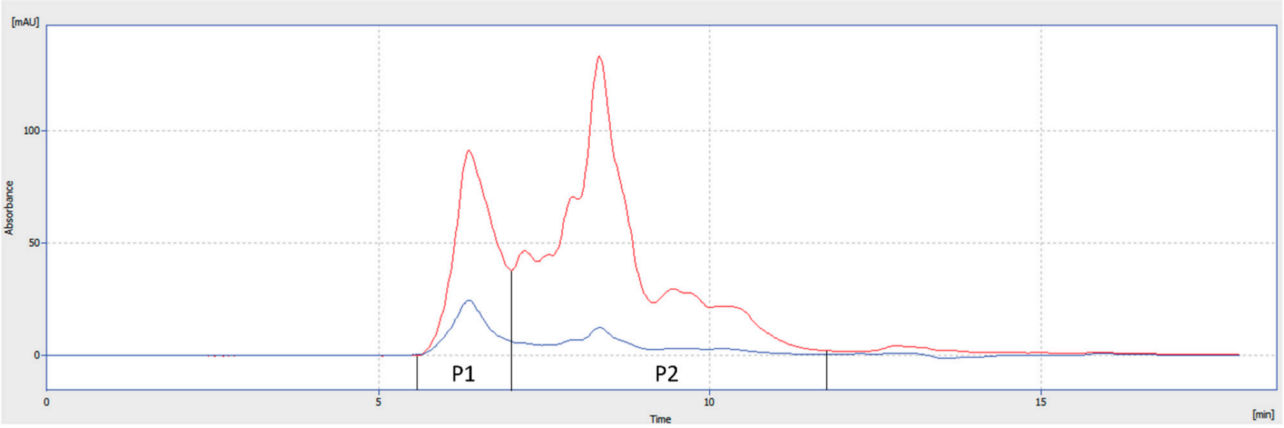

Figure S6. Chromatograms of SE-HPLC. Example of chromatograms obtained from the SE-HPLC analysis of the soluble (in red) and the insoluble (in blue) fractions. These have been used for %UPP calculation. The graph was obtained using the software Clarity v.8.8 (DataApex, Prague, Czech Republic).
